# Stroke risk assessment in children with sickle cell disease using transcranial Doppler ultrasound in Cameroon

**DOI:** 10.11604/pamj.2025.52.159.49120

**Published:** 2025-12-16

**Authors:** Sylviane Dongmo Fomekong, Yanelle Wandji, Marlyse Epotto, Joshua Tambe, Yolande Djike Puepi Fokam, Jean Roger Moulion Tapouh, Micheal Nko’o Amvene, Charlotte Eposse Ekoube, Boniface Moifo

**Affiliations:** 1 Department of Internal Medicine and Pediatrics, Faculty of Health Sciences, University of Buea, Buea, Cameroon,; 2 Medical Imaging Center, Regional Hospital of Buea, Buea, Cameroon,; 3 Département de Radiologie et d´Imagerie Médicale, Faculté de Médecine et des Sciences Pharmaceutiques, Université de Dschang, Dschang, Cameroun,; 4 Département de Radiologie et d´Imagerie Médicale, Faculté de Médecine et des Sciences Biomédicales, Université de Yaoundé I, Yaoundé, Cameroun,; 5 Département des Sciences Cliniques, Faculté de Médecine et des Sciences Pharmaceutiques, Université de Douala, Douala, Cameroun,; 6 Centre Intégré de Prise en Charge de la Drépanocytose, Hôpital Laquintinie de Douala, Douala, Cameroun

**Keywords:** Sickle cell disease, stroke, cerebral vasculopathy, cerebral arterial velocity, transcranial Doppler ultrasound, Cameroon

## Abstract

**Introduction:**

Stroke is a severe complication of sickle cell disease, often resulting from cerebral vasculopathy. Transcranial Doppler Ultrasound is a validated tool for stroke risk prediction in sickle cell disease, enabling timely preventive interventions. This study assessed cerebral vasculopathy in children with sickle cell disease using transcranial Doppler in three hospitals in Cameroon.

**Methods:**

a cross-sectional study was conducted from January to April 2024 in Buea, Limbe, and Laquintinie Hospitals. Children aged 2-16 years with confirmed sickle cell disease were evaluated. Data on demographics, clinical history, immunization status, hematological parameters, and cerebral blood flow velocities (time-averaged mean maximum velocity and peak systolic velocity in the middle cerebral and distal internal carotid arteries) were collected. Stroke risk was classified per the stroke prevention trial for sickle cell anemia (STOP) trial criteria. Statistical analysis included descriptive, bivariate, and multivariate methods (SPSS v23.0), with significance set at p<0.05.

**Results:**

among 110 participants (mean age 8.35 ± 3.74 years), 79.1% had normal arterial velocities, 12.7% intermediate, and 8.2% high velocities, indicating elevated stroke risk. Acute chest infections could be significantly associated with high velocities, while prophylactic vaccination (meningococcal and pneumococcal) could be protective.

**Conclusion:**

approximately 8.2% of children with sickle cell disease showed high cerebral artery velocities, suggesting high stroke risk. Acute chest infections may exacerbate this risk, whereas vaccination could appear protective. Transcranial Doppler is a valuable tool for early stroke risk stratification in pediatric sickle cell disease populations in resource-limited settings.

## Introduction

Sickle cell disease (SCD) is one of the most common inherited blood disorders in the world, affecting millions of people [1]. Current estimates suggest that approximately 4.4 million people live with SCD worldwide, while an additional 43 million carry the sickle cell trait [2]. The disease is most prevalent among people whose ancestors came from sub-Saharan Africa, the Mediterranean region, the Arabian Peninsula and India [1,3]. In the United States alone, around 100,000 individuals are affected [4]. The World Health Organization (WHO) has recognized its global significance, considering SCD as a major public health concern, particularly in Africa [2].

Sub-Saharan Africa bears the highest burden of SCD [2], with childhood mortality rates ranging from 50% to 90% among children homozygous for hemoglobin S [5]. It is estimated that 240,000 children are born with SCD annually in sub-Saharan Africa, and 50% to 80% of these children die before reaching 5 years of age [5]. The prevalence of sickle cell trait varies markedly between different countries but reaches levels as high as 40% in some areas of sub-Saharan Africa, Eastern Saudi Arabia, and central India [6]. In Cameroon, the trait is present in approximately 18.2% of the population in its heterozygous form, and 2-3% in its homozygous form [7].

The main clinical manifestations of SCD are related to anemia and vaso-occlusive events [8,9]. It is also one of the leading hematologic conditions associated with neurological complications in children. Notably, up to 25% of affected children may experience their first cerebral infarct before the age of six [10]. Stroke is one of the deadliest and most disabling complications for children with SCD [1]. Adams *et al*. reported SCD as the most common cause of childhood stroke occurring in up to 11% of children with SCD and peaking between the ages of 2 and 9 years [11]. The incidence of subsequent stroke is between 50% and 90% within 3 years of the first event. Medium-sized arteries of the circle of Willis, including the carotid arteries, are particularly vulnerable to the effects of sickled red blood cells and chronic hemolysis, resulting in stenosis and formation of fragile collaterals [1]. Sickle cell patients are at risk of silent cerebral infarcts and both ischemic and hemorrhagic strokes. Ischemic strokes occur mostly in young children and adults, with their highest incidence in children aged 2-16 years, while hemorrhagic strokes occur mostly in young adult patients (20-29 years) [12].

Neurological complications, particularly stroke, can cause significant disability with important socioeconomic and psychological impacts on sickle cell patients (SCP) and their families, and can even lead to death if not properly managed [13]. The Stroke Prevention Trial in Sickle Cell Anemia (STOP), reported by Nichols *et al*. demonstrated that regular blood transfusions significantly reduce the risk of stroke in children identified as high-risk through transcranial Doppler ultrasound (TCD) screening [14]. TCD ultrasound is a sensitive and specific tool for detecting cerebral vasculopathy in patients with SCD [9]. The Brazilian guidelines of stroke prevention say TCD ultrasound should be used as a method for primary prevention of stroke in SCP aged between two and sixteen years of age [15]. Early implementation of TCD screening from the age of two has been shown to substantially reduce the incidence of first-time strokes in this population [16]. The aim of this study is to characterize the cerebral arterial velocity profiles on transcranial Doppler (TCD) in children with sickle cell anemia, determine the prevalence of high-risk velocities, and identify associated factors.

## Methods

**Study area, design, and period:** this was a hospital-based cross-sectional study carried out in the Laquintinie Hospital of Douala and the Buea and Limbe Regional Hospitals. Data were collected from January 2024 to April 2024.

**Study population, inclusion, and exclusion criteria:** the study included all consenting and or assenting sickle cell patients aged between 2 years and 16 years with a confirmed diagnosis (sickle cell anemia-hemoglobin SS) and being followed in selected hospitals. SCPs with inadequate acoustic windows and those with incomplete TCD examinations were excluded.

**Sample size and sampling technique:** a minimum of 97 participants was required. This was calculated using the COCHRAN formula as follows:


$$n=\frac{Z^{2}pq}{E^{2}}$$

Where n is the sample size; Z is the z-score for the confidence level; p is the estimated population proportion; q is 1-p; and e is the margin of error. Taking a 0.05 margin of error at a 95% confidence level. The prevalence of stroke in SCD in Kenya was studied by Uyoga *et al*. in 2019 [17]. A convenient sampling method was used.

**Data collection:** data were collected by the main investigator through an interviewer-administered pre-structured and pre-tested data collection form. The data collection form was written in English but was translated into local languages for non-English speaking participants. Participants were met before the transcranial Doppler ultrasound, consent and or assent were obtained, and the following data were collected: sociodemographic data, clinical and hematologic data. TCD hemodynamic data were also collected. Participants had their transcranial Doppler ultrasound done free of charge by two radiologists, doctors having more than five years of experience each. Two ultrasound machines were used: a Sonoscape S8 portable machine in service since 2016 with Doppler phase array probe (2-5MHz) for participants from the Buea and Limbe Regional Hospitals. A portable ultrasound machine, VIVID I, manufactured by General Electric, with a phase array probe (2-5MHz) in service since 2009, was used for participants from Laquintinie hospital. All patients´ information was coded to ensure confidentiality and anonymity.

**Study variables:** independent variables were sociodemographic data (age, gender), clinical data (age of diagnosis, history of stroke, daily water intake, medications taken, frequency of transfusions and hospital admissions), hematologic data (white blood and red blood cell counts, hematocrit, mean cell volume, mean cell hemoglobin and mean cell hemoglobin concentration).

Dependent variables were TCD hemodynamic data, including the peak systolic velocities (PSV) and time-averaged mean maximum velocities (TAMMV) in both the proximal middle cerebral artery (MCA) and the distal internal carotid arteries (ICA) of both sides of the head. Mean averages of the TAMMV and PSV were recorded, and the highest TAMMV was used to classify participants as per the stroke prevention trial in sickle cell anemia (STOP Criteria) [14] into no risk (<170 cm/s), intermediate risk (170-200 cm/s), and high risk (>200 cm/s) for stroke. PSV is a spectral Doppler index measured by Doppler ultrasonography, which represents the initial peak of each cardiac cycle´s TCD waveform [18]. TAMMV refers to the time mean of the peak velocity envelope; the envelope being a trace of the peak flow velocity as a function of time [16].

**The transcranial Doppler examination procedure:** the procedure was done with the patient in supine, with lateral tilting of his head to either side using the trans-temporal approach. Participants were scanned when well relaxed, but not asleep. The phase array probe was placed on the temporal aspect of the head, cephalad to the zygomatic arch and immediately anterior and slightly superior to the tragus of the ear in the transverse position. Then the probe was angulated anteriorly till the M1 segment of the middle cerebral artery was visualized on a color Doppler. Then, spectral wave analysis was done in the M1 segment on both sides. The trans-temporal view can examine the internal carotid artery bifurcation at the underlined depths with flow. The internal carotid artery was identified at depths of 55-65 mm with the simultaneous flow toward or away from the probe [18] on both sides of the participant´s head.

**Data management and analysis:** the data collected was keyed into the census and survey processing system (CSPro) version 7.7, from where it was exported to an Excel document. Data were analyzed using the Statistical Package Social Sciences (SPSS) version 23.0. Frequencies, tables, and percentages were used for categorical variables. Descriptive statistics (mean and standard deviation) were used for numerical variables. Chi-square was used to analyze categorical variables (gender). Bivariate analysis was used to check for associations between clinical and hematologic factors with high cerebral arterial velocities. The multiple logistic regression was used to control for confounders between clinical, immunological, and hematological factors with high cerebral arterial velocities. The confidence interval was 95% and a p-value < 0.05 was considered statistically significant.

**Ethical clearance:** an ethical clearance, number 2023/2210-11/UB/SG/IRB/FHS, was obtained from the institutional review board of the Faculty of Health Sciences (FHS) of the University of Buea. Administrative approval was obtained from the South West Regional Delegation and from the directors of the three hospitals. Prior to data collection, written informed consent and or assent was obtained from respective participants.

## Results

**Sociodemographic characteristics of participants:** out of 110 participants included in this study, 62 were male (56.4%) with a sex ratio of 1.3: 1. The mean age of participants was 8.35 ± 3.74 years, with the age group 6 to 11 years being the most represented, n=54 (49.1%).

**Description of clinical, immunological, and hematological factors within the study population:** we found that 94.5% (104) of participants were on daily folic acid. About 51% (56) received prophylactic vaccines against encapsulated germs. A recent acute chest infection was reported for about 12.7% (14) of participants, and about 43.3% (45) of them were taking hydroxyurea daily. Most participants (90%) were following high daily water intake therapy. The description of hematologic factors represented in Table 1 shows that the mean hemoglobin level and hematocrit levels were 7.7 ± 1.5 g/dL and 24.4 ± 4.7%, respectively.

**Table 1:** description of hematologic factors among study participants

Variable	Frequency (n=110)	Mean ± SD
WBC (cells/mm^3^)	102	14.4 ± 5.9
RBC (cells/mm^3^)	102	3.1 ± 3.5
Hb level (g/dL)	77	7.7 ± 1.5
Hematocrit (%)	102	24.4 ± 4.7
MCV (Fl)	102	86.3 ± 9.8
MCH (pg)	102	29.3 ± 3.6
MCHC (g/dL)	102	33.0 ± 2.3

WBC: white blood cell count; RBC: red blood cell count; Hb: hemoglobin; MCV: mean cell volume; MCH: mean cell hemoglobin; MCHC: mean cell hemoglobin concentration; SD: standard deviation

**Figure 1:**
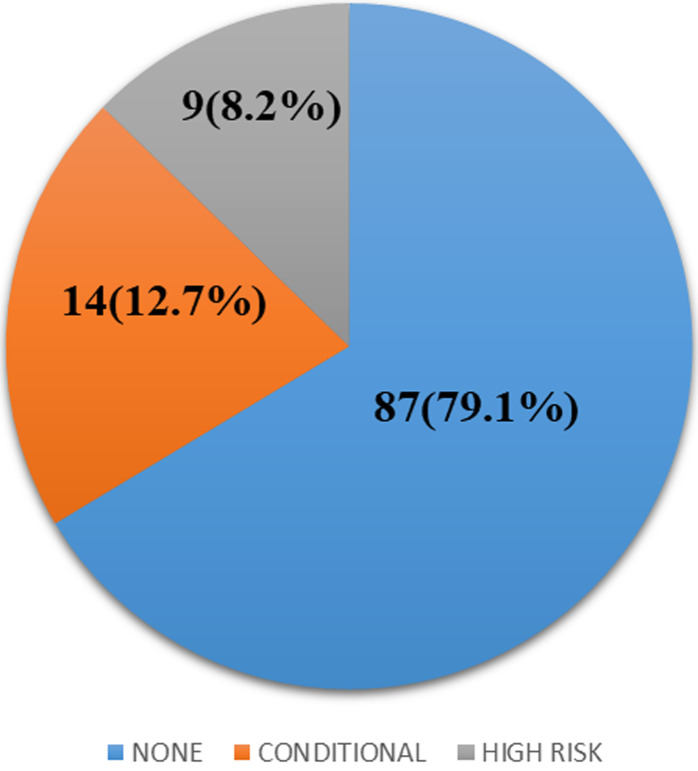
classification of participants according to stroke risk

**Prevalence of sickle cell anemia children with high cerebral arterial velocities:** based on the STOP criteria using the TAMMV, out of 110 participants, 87(79.1%) were classified as no risk of stroke, 14 (12.7%) as conditional risk for stroke and 9 (8.2%) as high risk for stroke (Figure 1).

**Distribution of stroke risk according to sociodemographic data:** the distribution of participants with high risk of stroke (n=9) showed that 6 (66.7%) came from Laquintinie hospital, 4 (44.7%) were from the 6 to 11 years age group, and females were predominant (55.6%).

**Association of clinical, immunological, and hematologic factors with high cerebral arterial velocities on bivariable analysis:** bivariable analysis was performed to analyze possible association between hematologic factors, including high steady state leucocytes, low red blood cell count, low hemoglobin, low hematocrit, low mean cell volume, low mean corpuscular hemoglobin, and low mean corpuscular hemoglobin concentration. Among these, none was found to be significantly associated with high cerebral arterial velocities (Table 2).

**Table 2:** association between hematologic factors and high risk of stroke on bivariable analysis

Independent variable		High risk of stroke		Chi-square value	p-Value
		Yes	No		
High WBC	Yes	1(11.1%)	19(18.8%)	0.329	0.566
	No	8(88.9%)	82(81.2%)		
Low RBC	Yes	5(55.6%)	49(48.5%)	0.164	0.686
	No	4(44.4%)	52(51.5%)		
Low Hb	Yes	5(55.6%)	32(33,3%)	1.914	0.167
	No	4(44.4%)	68(67.3%)		
Low Hct	Yes	1(11.1%)	5(5.0%)	0.608	0.435
	No	8(88.9%)	96(95.0%)		
Low MCV	Yes	2(22.2%)	28(27.7%)	0.126	0.723
	No	7(77.8%)	73(72.3%)		
Low MCH	Yes	1(11.1%)	24(23.8%)	0.753	0.385
	No	8(88.9%)	77(76.2%)		
Low MCHC	Yes	0(0%)	9(8.9%)	0.873	0.350
	No	9(100%)	92(91.1%)		

WBC: white blood cell count; RBC: red blood cell count; Hb: hemoglobin level; Hct: hematocrit level; MCV: mean cell volume; MCH: mean corpuscular hemoglobin; MCHC: mean corpuscular hemoglobin concentration

**Figure 2:**
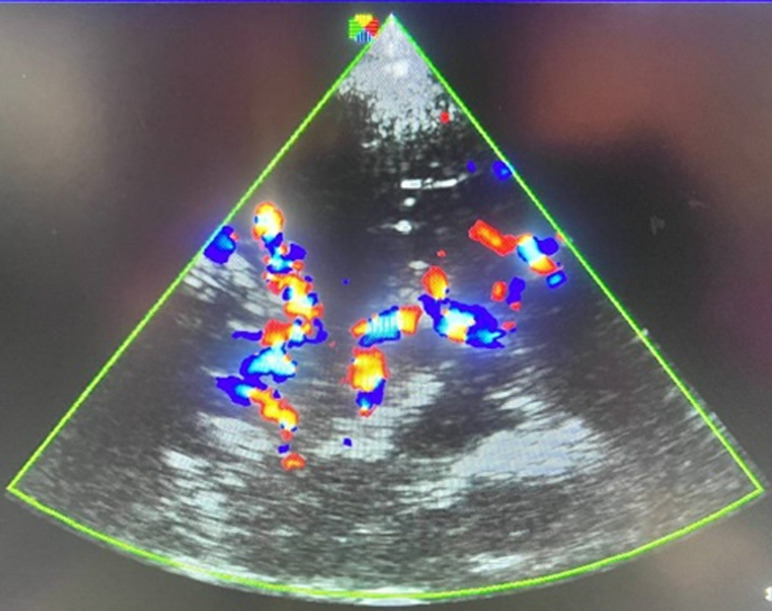
structure of the circle of Willis on color Doppler ultrasound

**Figure 3:**
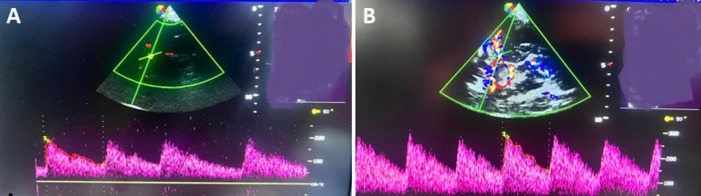
examples of transcranial Doppler ultrasound spectrum: A) left internal carotid artery waveform showing normal velocities with pSV 176 cm/s and TAMMV of 115 cm/s; B) left internal carotid artery waveform showing high PSV 217 cm/s and high intermediate TAMMV 191 cm/s

**Association of clinical, immunological, and hematologic factors with high cerebral arterial velocities on multivariable analysis:** multivariable analysis was performed simultaneously, taking into account all the variables selected in the bivariate analysis. After fitting a multiple logistic regression x (adjusted odds ratio, confidence interval p value), recent acute chest infection (adjusted odds ratio=7.36 at 95% confidence interval=1.26-42.99 p=0.027) predisposes up to seven times to high cerebral arterial velocities and taking prophylactic vaccines (adjusted odds ratio=0.07 at 95% confidence interval=0.01-0.67 p value=0.021) was found to protect against high cerebral arterial velocities (Table 3, Figure 2, Figure 3).

**Table 3:** association between clinical, immunological factors and high risk of stroke on multivariable analysis

Independent variable	Odds ratio at 95% confidence interval	Adjusted odds ratio at 95% confidence interval	p-value
**Recent chest infection**			
Yes	4.091(0.894-18.718)	7.36(1.26-42.99)	0.027
No	1	1	
**Prophylactic vaccines**			
Yes	0.105(0.013-0.867)	0.07(0.01-0.67)	0.021
No	1	1	

## Discussion

This study aimed to describe the velocity profile of cerebral arteries on TCD of SCP, to determine the prevalence of SCP with high cerebral arterial velocities, and to determine factors associated with these. Among the 110 participants, 9 (8.2%) presented with elevated cerebral arterial velocities, indicating a high risk of stroke. Taking prophylactic vaccines against meningococcal and pneumococcal infections (p=0.013) was found to protect against high cerebral arterial velocities, whereas recent acute chest infection (p=0.053) was a risk factor.

**Classification of participants according to risk of stroke:** based on TCD results, 87 participants (79.1%) were classified as having no stroke risk, 14 (12.7%) as conditional risk, and 9 (8.2%) as high risk. These proportions are consistent with studies conducted in other countries with comparable sickle cell anemia prevalence [1,19,20]. However, our findings differ from those of Thurn *et al*. (2022) in Regensburg, Germany, where 50% of the 26 participants had abnormal velocities [21]. This discrepancy may be attributed to differences in sample size, population characteristics, and regional disease burden. Our study involved a larger cohort from a high-risk population, which strengthens the generalizability of our results within similar contexts.

**Distribution of stroke risk according to the sociodemographic and hematological characteristics of participants:** analysis of stroke risk distribution revealed that most high-risk participants (66.7%) were from the Laquintinie Hospital, which hosts a dedicated sickle cell management center. This suggests that specialized care centers may attract more severe cases or facilitate better detection. The majority of participants belonged to the 2-5 years age group (44.7%), consistent with literature indicating that stroke risk peaks in early childhood due to progressive vasculopathy [10-12]. As for the distribution of stroke risk according to hematologic factors, the mean value of hemoglobin was low (hemoglobin=7.47 ± 1.51 g/dL) in children classified under high risk of stroke, but the difference was not significant. This was similar to results obtained by Dorie *et al*. (7.45 ± 1.2 g/dL), suggesting a potential trend that warrants further investigation [20].

**Factors associated with high cerebral arterial velocities:** bivariable analysis did not reveal any significant associations between hematologic factors and elevated cerebral velocities. This contrasts with findings from Ekoube *et al*. in 2021, who identified high steady-state leukocyte counts (p=0.001) and low hemoglobin levels (p<0.001) as significant predictors of stroke risk [22]. The divergence may reflect differences in study design, population characteristics, or statistical power. Multivariable logistic regression analysis provided more nuanced results. A recent acute chest infection was found as a potential risk factor (adjusted odds ratio [AOR] = 7.36; 95% CI: 1.26-42.99; p=0.027), indicating that affected children were over seven times more likely to have high cerebral arterial velocities. This aligns with Ekoube *et al*. findings, reinforcing the role of acute inflammatory episodes in exacerbating cerebrovascular risk [22].

Conversely, receiving prophylactic vaccines against meningococcal and pneumococcal infections was significantly protective (AOR = 0.07; 95% CI: 0.01-0.67; p=0.021). This highlights the importance of preventive healthcare measures against stroke risk, possibly by reducing infection-related inflammation and vascular stress.

**Limitations:** data on reticulocyte counts and hemoglobin sub-genotypes could not be obtained. Our study was conducted in a short period of time; a cohort study over a longer period of time would have been more appropriate to evaluate risk factors associated with stroke.

## Conclusion

There is a high risk of stroke of about 8.2% in our study population of children suffering from sickle cell disease, according to the STOP criteria. A recent acute chest infection was found to be associated with high cerebral arterial velocities, and taking prophylactic vaccines could protect against high cerebral arterial velocities. Transcranial Doppler ultrasound should be integrated in the routine follow-up of sickle cell anemia patients as recommended by the STOP criteria.

### What is known about this topic

- High stroke risk in children with SCD, especially silent strokes due to progressive cerebral vasculopathy; the highest risk occurs between ages two and nine, with up to 11% of children affected by overt stroke if no preventive measures are taken;
- Transcranial Doppler (TCD) is a validated, non-invasive screening method for detecting elevated cerebral arterial velocities, which are predictive of stroke risk in children with SCD; yearly TCD screening, from age two, has been shown to reduce the incidence of first stroke when paired with prophylactic interventions such as chronic transfusion therapy [STOP Trial].

### What this study adds

- This study identifies the proportion of high risk of stroke in a sickle cell population in Cameroon;
- It highlights prophylactic vaccines as a potential protective factor against cerebral vasculopathy and recent chest infection as a risk factor for high cerebral arterial velocities.
